# Publisher Correction: FGF signalling controls the specification of hair placode-derived SOX9 positive progenitors to Merkel cells

**DOI:** 10.1038/s41467-018-05361-8

**Published:** 2018-07-17

**Authors:** Minh Binh Nguyen, Idan Cohen, Vinod Kumar, Zijian Xu, Carmit Bar, Katherine L. Dauber-Decker, Pai-Chi Tsai, Pauline Marangoni, Ophir D. Klein, Ya-Chieh Hsu, Ting Chen, Marja L. Mikkola, Elena Ezhkova

**Affiliations:** 1 Black Family Stem Cell Institute, Department of Cell, Developmental, and Regenerative Biology, Icahn School of Medicine at Mount Sinai, 1 Gustave L. Levy Place, New York, NY 10029 USA; 2 Developmental Biology Program, Institute of Biotechnology, University of Helsinki, 00014 Helsinki, Finland; 3 National Institute of Biological Sciences, 102206 Beijing, China; 4 Department of Stem Cell and Regenerative Biology, Harvard Stem Cell Institute, Harvard University, Cambridge, MA 02138 USA; 5 Department of Orofacial Sciences and Program in Craniofacial Biology, University of California San Francisco, San Francisco, CA 94143 USA; 6 Department of Pediatrics and Institute for Human Genetics, University of California San Francisco, San Francisco, CA 94143 USA

Correction to: *Nature Communications* ; 10.1038/s41467-018-04399-y, published online 13 June 2018.

The originally published version of this Article contained an error in Figure 2. In panel e, the blue bar was incorrectly labelled ‘KRT8(+)/TOMATO(−)’. Furthermore, during the process of preparing a correction, the publication date of the Article was inadvertently changed to June 20^th^ 2018. Both of these errors have been corrected in the PDF and HTML versions of the Article.

