# Supplementary material for: FGF signalling controls the specification of hair placode-derived SOX9 positive progenitors to Merkel cells
Source: Nat Commun. 2018 Jun 20;9:2333. doi: 10.1038/s41467-018-04399-y (PMC5998134; doi:10.1038/s41467-018-04399-y)
Supplement: Supplementary file 1 — Supplementary Information [file 41467_2018_4399_MOESM1_ESM.pdf]

Supplementary Information

FGF signalling controls the specification of hair placode-derived SOX9 positive progenitors to Merkel cells

Nguyen et al.

Supplementary Figure 1

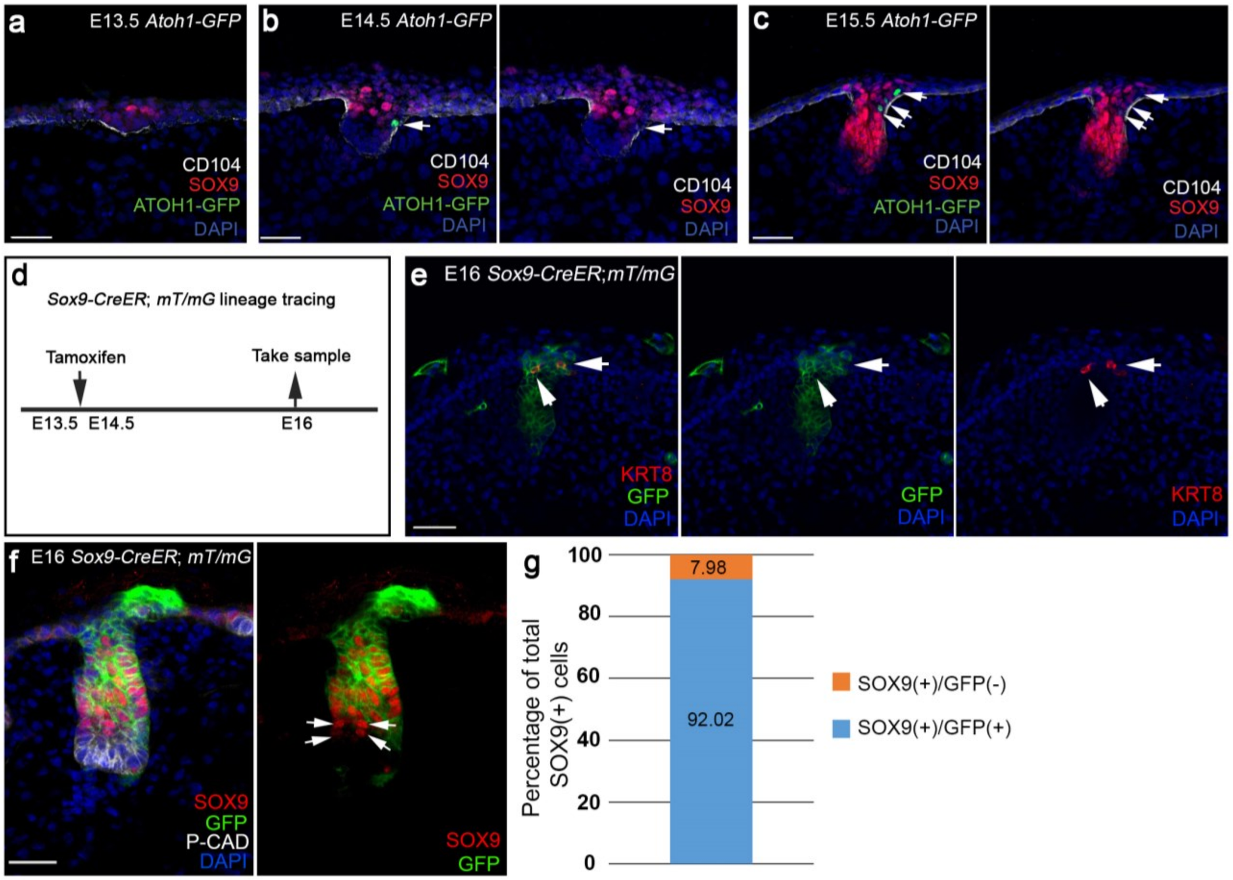

Supplementary Figure 1. Merkel cells originate from SOX9-expressing cells inside hair placode structures at E16. (**a-c**) Immunofluorescence staining for SOX9 (red) and early Merkel cell specification marker ATOH1 (green) shows no overlap at E13.5 (a), E14.5 (b), and E15.5 (c); arrows point to Merkel cells. (**d**) Experimental design for *Sox9-CreER*: *mT/mG* lineage tracing. (**e**) Immunofluorescence analysis of KRT8 in *Sox9-CreER*; *mT/mG* lineage tracing at E16. SOX9-traced cells are GFP(+) (green). KRT8(+) Merkel cells are stained in red. Note the overlap between SOX9 progenies and KRT8 staining (white arrows). (**f**) Immunofluorescence analysis of SOX9 in *Sox9-CreER*; *mT/mG* lineage tracing at E16. SOX9-traced cells are GFP(+) (green). SOX9(+) cells are stained in red. Arrows point to SOX9(+) cells that are not SOX9-traced cells, indicating incomplete recombination. Scale=50µm in a, b, c, e, and f. (**g**) Quantifications of SOX9-traced (GFP(+)) and -untraced (GFP(-)) SOX9(+) cells in E16 *Sox9-CreER*; *mT/mG* skins. n=5. Data is presented as a percentage of the total number of cells quantified. Bar graph shows the percentage of SOX9(+) cells which are co-labelled with GFP (blue) or not co-labelled with GFP (orange).

Supplementary Figure 2

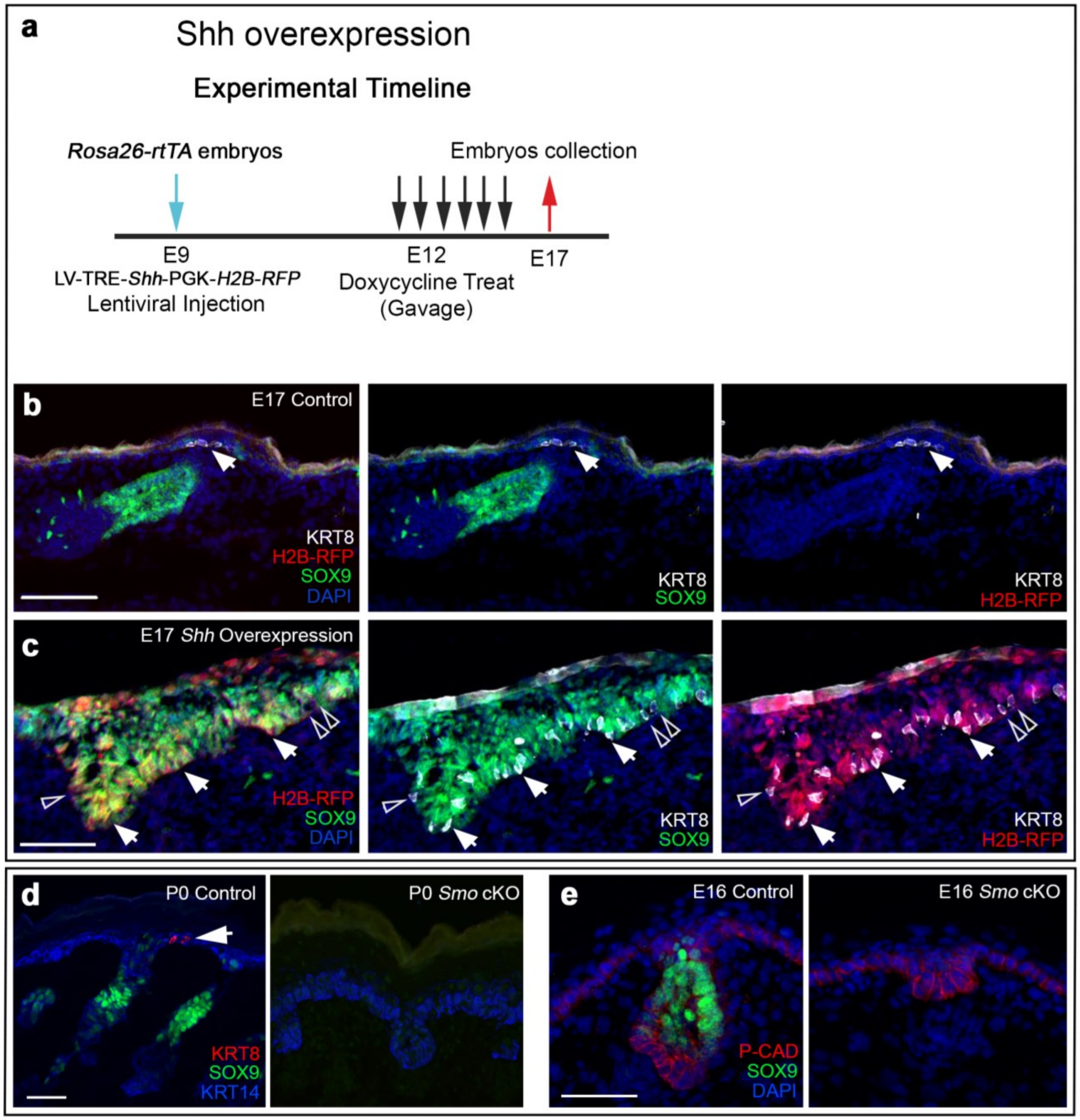

Supplementary Figure 2. SHH signalling promotes the specification of SOX9(+) cells in the developing skin. (**a-c**) Doxycycline-inducible Shh overexpression in the skin epithelium. (**a**) Experimental design for Shh overexpression. High-titer lentiviruses expressing a doxycycline-inducible *Shh*-PGK-*H2B*-RFP construct were injected into the amniotic fluid of E9 *Rosa26-rtTA* embryos to infect the epidermis; doxycycline treatment from E12-E17 induced the expression of SHH and H2B-RFP proteins in the skin epithelium, and samples were collected at E17 for Immunofluorescence analysis. (**b**) Immunofluorescence staining for SOX9 (green) and KRT8 (white) in control; arrows point to Merkel cells. (**c**) Immunofluorescence staining for SOX9 (green) and KRT8 (white) in Shh-overexpressing embryos at E17. Note the altered morphology of skin epithelium overexpressing Shh and the ectopic expression of SOX9 and KRT8. Arrows indicate KRT8(+) cells that are co-labelled with SOX9 (middle panel) or H2B-RFP (right panel). Open arrowheads indicate KRT8(+) cells that are not co-labelled with SOX9 (middle panel) or H2B-RFP (right panel) (c). (**d**) Immunofluorescence staining for SOX9 (green) and KRT8 (red) in P0 control and *Smo* cKO back skins. Arrow points to Merkel cells. Note the loss of KRT8(+) cells in *Smo* cKO skin. (**e**) Immunofluorescence staining for SOX9 (green) in *Smo* cKO and control placodes at E16. Note the loss of SOX9(+) and KRT8(+) cells in *Smo* cKO skin. Scale=50μm in b, c, d, and e.

Supplementary Figure 3

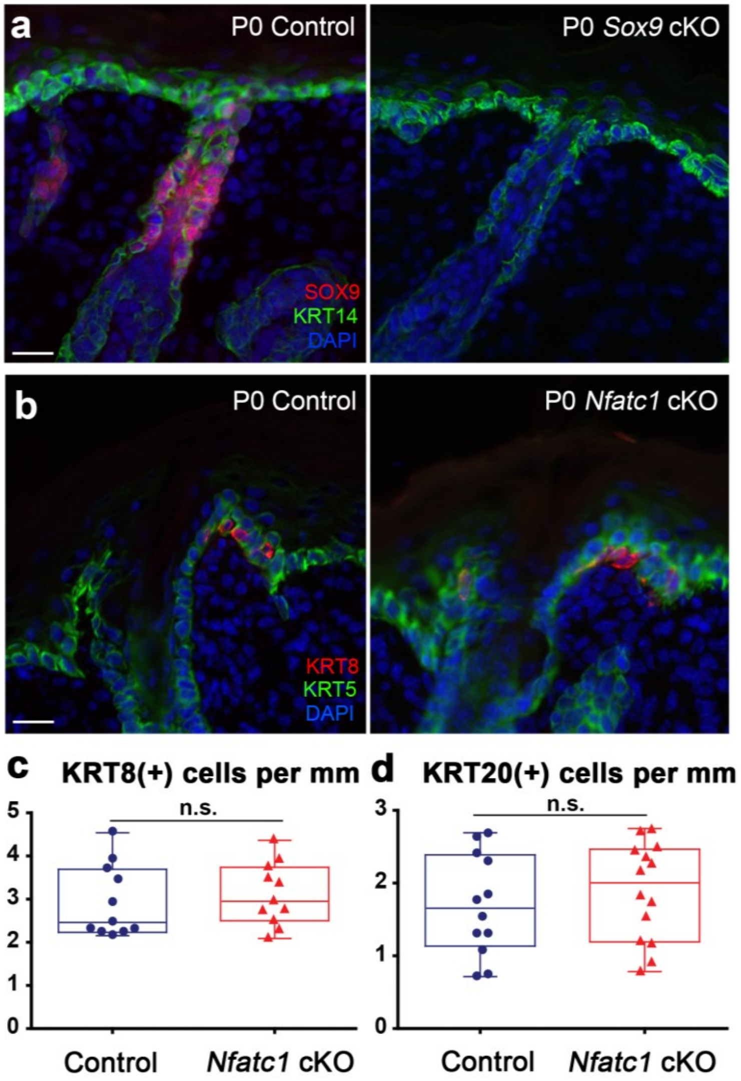

Supplementary Figure 3. Transcription factors SOX9 and NFATc1 are dispensable for Merkel cell formation. (**a**) Immunofluorescence staining for SOX9 (red) in control and *Sox9* cKO mice, confirming the loss of SOX9 expression in *Sox9* cKO back skin. (**b**) Immunofluorescence staining for KRT8 (red) in control and *Nfatc1* cKO back skin. (**c**) Quantification of KRT8(+) Merkel cells in control and *Nfatc1* cKO back skins at P0. n=3, p=0.5113. (**d**) Quantification of KRT20(+) Merkel cells in control and *Nfatc1* cKO back skins at P0. n=3, p =0.5036. Scale=20μm in a and b. The data presented in box plots (c, d) shows the median with 25th and 75th percentile borders. Whiskers extend from minimum to maximum. n.s., not significant (the Mann-Whitney test).

Supplementary Figure 4

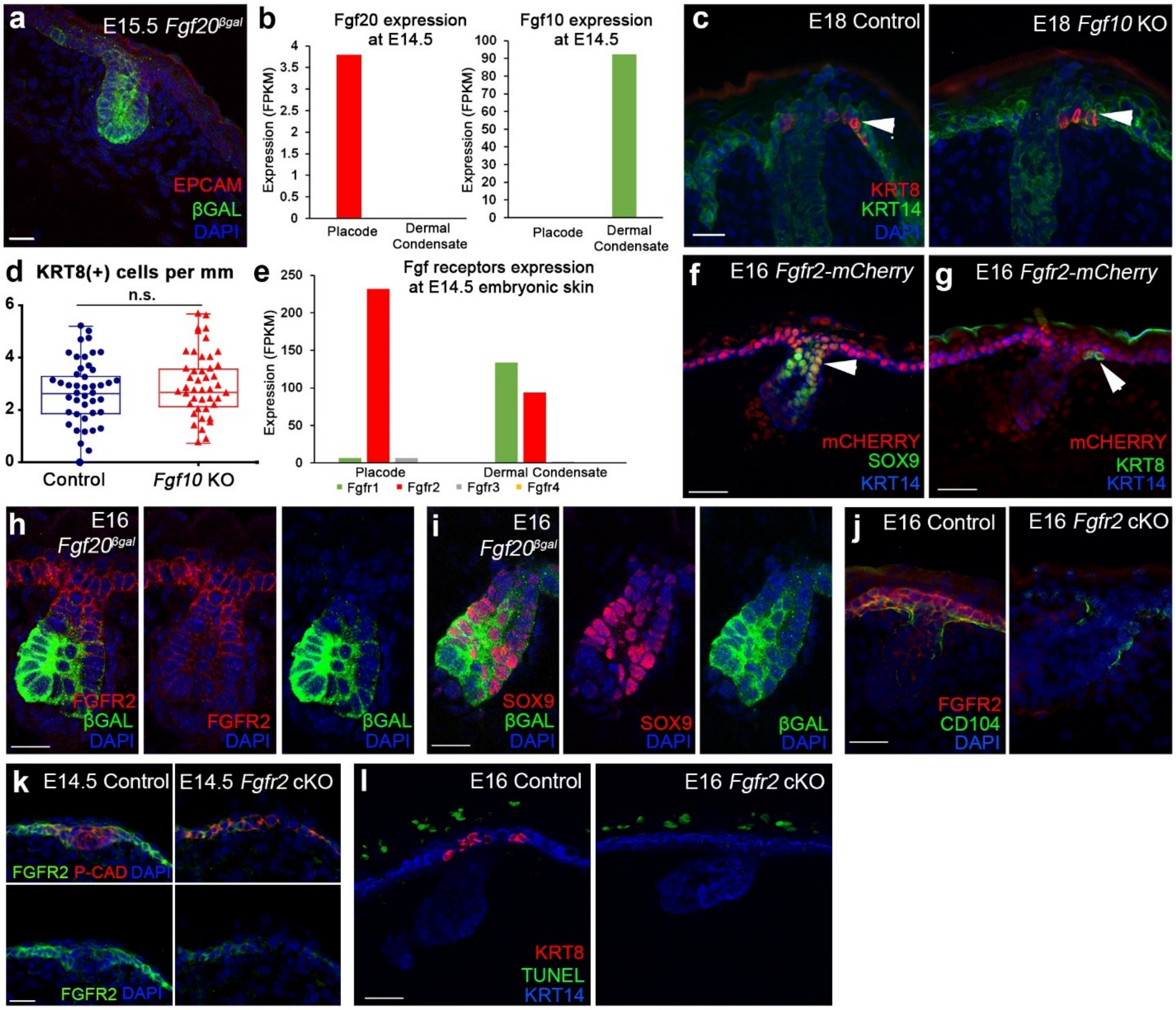

Supplementary Figure 4. Expression of components of FGF signalling in embryonic skin. (**a**) Analysis of *Fgf20*$^{\beta gal}$ reporter mice shows that Fgf20 is expressed in developing hair follicles. (**b**) Expression of Fgf20 (left bar chart) and Fgf10 (right bar chart) in the placode and dermal condensate at E14. Data is presented as the mean expression in FPKM values obtained from RNA-seq data[35][http://hair-gel.net]. (**c**) Immunofluorescence staining for KRT8 (red) and KRT14 (green) in *Fgf10* KO and control back skins at E18. Arrows point to Merkel cells. Scale=20μm. (**d**) Quantification of KRT8(+) Merkel cells in *Fgf10* KO and control back skins at E18. n=5, p=0.8704. The data presented in box plots show the median with 25th and 75th percentile borders. Whiskers extend from minimum to maximum. n.s., not significant (the Mann-Whitney test). (**e**) Expression of Fgf receptors 1-4 in the placode and dermal condensate at E14. Data are presented as the mean expression in FPKM values obtained from RNA-seq data[35] [http://hair-gel.net]. (**f**) Immunofluorescence staining for SOX9 (green) in *Fgfr2-mCherry* transgenic mice. Arrow points to cells positive for both SOX9 and mCHERRY. (**g**) Immunofluorescence staining for KRT8 (green) in *Fgfr2-mCherry* transgenic mice. Arrow points to Merkel cells. (**h-i**) Immunofluorescence analysis of *Fgf20*$^{\beta gal}$ reporter mice at E16. (**h**) Immunofluorescence staining for FGFR2 (red) and βGAL (green). (**i**) Immunofluorescence staining for SOX9 (red) and βGAL (green). (**j-k**) Immunofluorescence staining for FGFR2 (green) in *Fgfr2* cKO and control mice at E16 (j) and E14.5 (k). Note the complete loss of FGFR2 in *Fgfr2* cKO epithelium at E16, whereas residual FGFR2 is present at E14.5. (**l**) TUNEL staining shows no apoptosis in control and *Fgfr2* cKO hair follicle cells at E16. Note TUNEL (+) apoptotic cells in the suprabasal differentiated layer of the epidermis that are undergoing cornification. Scale=50μm in a, f, g h, i, j, k, and l.

Supplementary Figure 5

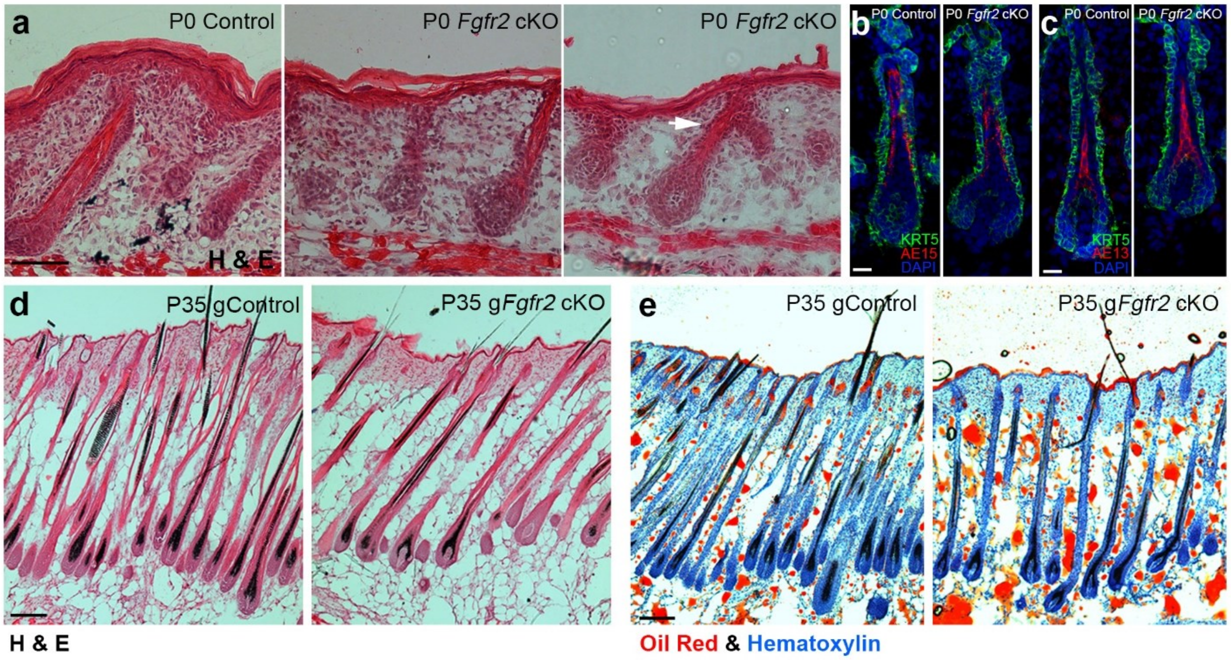

Supplementary Figure 5. FGFR2 is not required for HFSC specification. (**a**) Hematoxylin and eosin (H&E) analysis of P0 skins of *Fgfr2* cKO and control mice. A small number of *Fgfr2*-null hair follicles showed a split hair phenotype (indicated by arrow). (**b-c**) Immunofluorescence staining for hair follicle differentiation markers in *Fgfr2* cKO and control mice at P0. (**b**) AE15 (red) labels the IRS and medulla. (**c**) AE13 (red) marks the cuticle and cortex of the hair shaft. (**d**) H&E analysis of full-thickness grafted *Fgfr2* cKO (g*Fgfr2* cKO) and grafted control (gControl) skins at P35. (**e**) Oil Red O analysis of P35 g*Fgfr2* cKO and gControl skins demonstrates the formation of sebaceous glands (SG). Scale=100μm in a, d, and e. Scale=20μm in b and c.

Supplementary Table 1. Primers used for mice genotyping

Primer name	Sequence
K14 Cre F	CACGATACACCTGACTAGCTGGGTG
K14 Cre R	CATCACCCACAGGCTAGCGCCAACT
Lhx2 Cre F	CTGCCACGACCAAGTGACAGCAATG
Lhx2 Cre R	GCCTTCTCTACACCTGCGGTGCTAA
Sox9 Cre ER WT F	CTTCCATCCCGCAGACCCA
Sox9 Cre ER Mutant F	CGGGCTCTACTTCATCGCATTC
Sox9 Cre ER Common R	ACAAAGTCCAAACAGGCAGGGA
Fgfr2 flox1	GCAATAGCCTGGATTAGAGG
Fgfr2 flox2	CATCTCTGCCATACCAAGTC
Fgfr2 flox3	GAAATGGAAGCGTAGAGGTC
Sox9 WT F	GGGGCTTGTCTCCTTCAGAG
Sox9 WT R	TGGTAATGAGTCATACACAGTAC
Sox9 flox F	ACACAGCATAGGCTACCTG
Sox9 flox R	TGGTAATGAGTCATACACAGTAC
Atoh1 GFP F	GAAGGGCATTTGGTTGTCTCA
Atoh1 GFP R	AGGGTCAGCTTGCCGTAGGT
Smo WT F	CCACTGCGAGCCTTTGCGCTAC
Smo WT R	CCCATCACCTCCGCGTCGCA
Smo flox F	CTTGGGTGGAGAGGCTATTC
Smo flox R	AGGTGAGATGACAGGAGATC
Fgfr2 mCherry F	CTGTTGCTTACTCTGGAGCT
Fgfr2 mCherry R	GATACCCCAACTCCATTCAC
mT/mG WT F	CTGTTGCTTACTCTGGAGCT
mT/mG Common R	CGAGGCGGATCACAAGCAATAA
mT/mG F	CTCTGCTGCCTCCTGGCTTCT
Nfatc1 F	GGACAGTCTAAGGCCTGCTG
Nfatc1 R	ACCCCACATCCCAGAGTGA
Fgf20 LacZ F	CTGCATTCGCCTCGCCACCCTTGCTACACT
Fgf20 LacZ KO R	GGCCTTCCTGTAGCCAGCTTTCATCAACAT
Fgf20 LacZ WT R	GGATCTGCAGGTGGAAGCCGGTGCGGCAGT
Fgf10 WT	CATTGTGCCTCAGCCTTTCCC
Fgf10 Mutant	CACCAAAGAACGGAGCCGGTTG
Fgf10 Common	CGACAGTCTTCATTCTTGGTCCT
